# Correction: *In silico* guided structural and functional analysis of genes with potential involvement in resistance to coffee leaf rust: A functional marker based approach

**DOI:** 10.1371/journal.pone.0238967

**Published:** 2020-09-03

**Authors:** The PLOS ONE Staff

Fig 2 is incorrect and there is an error in its figure legend. The authors have provided corrected versions here.

**Fig 2:**
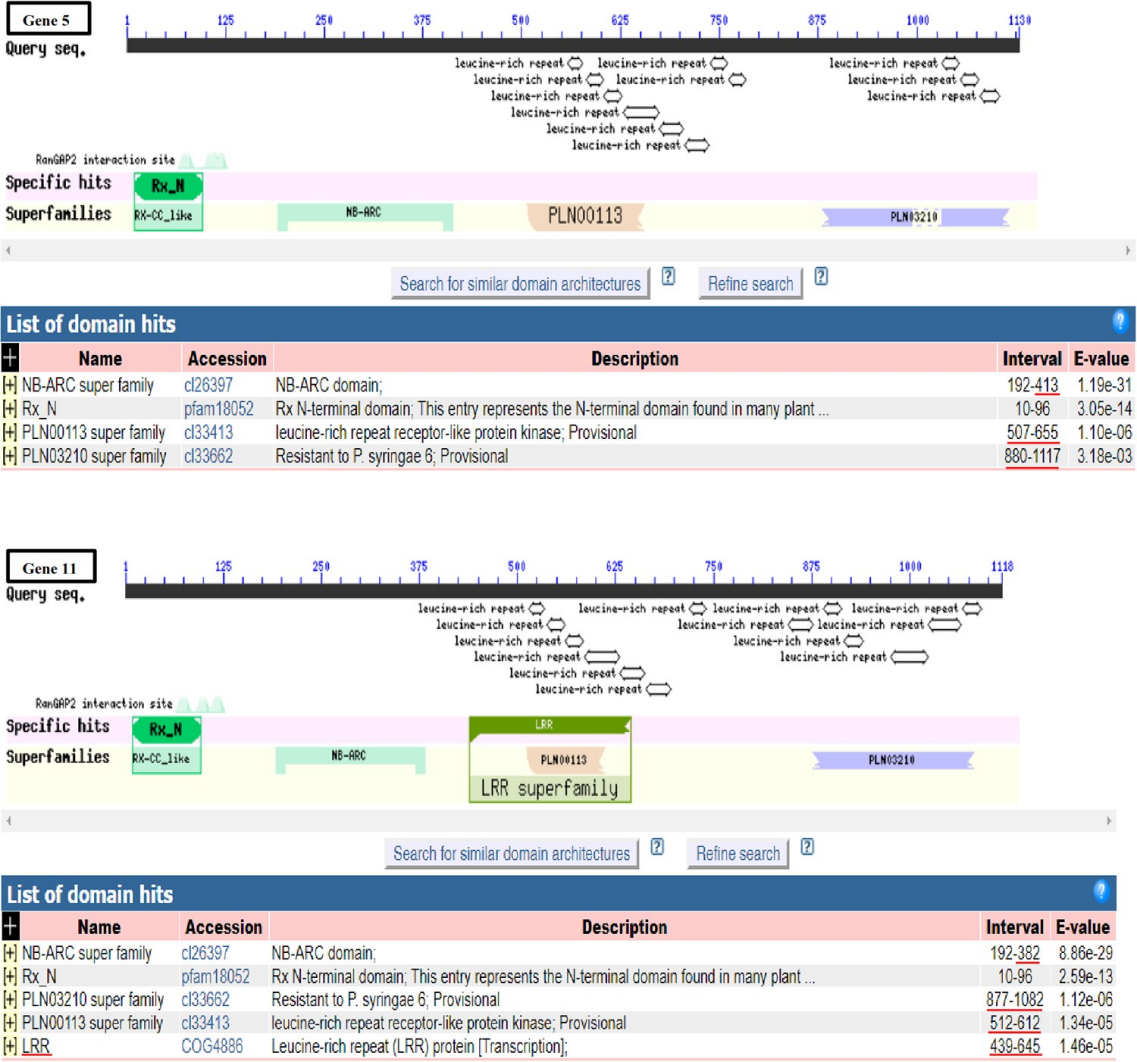
Comparison of conserved domains and motif architecture in genes 5 and 11. Different numbers of domain hits and variations in the domain length were detected and compared in both genes. Red bar (s) on the left side was (were) used to spot domain (s) exclusive to each gene while those on the right side were used to show the variation in the interval (number) of the amino acids constituting the respective domain and the polymorphisms in the size of the domains. Conserved domains were detected using NCBI Conserved Domain Database (https://www.ncbi.nlm.nih.gov/Structure/cdd/wrpsb.cgi). Graphical summary was set to standard view. https://doi.org/10.1371/journal.pone.0222747.g002.

## References

[1] Barka GD, Caixeta ET, Ferreira SS, Zambolim L (2020) *In silico* guided structural and functional analysis of genes with potential involvement in resistance to coffee leaf rust: A functional marker based approach. PLoS ONE 15(7): e0222747 10.1371/journal.pone.0222747 32639982 PMC7343155

