# Supplementary figures and images for: In silico guided structural and functional analysis of genes with potential involvement in resistance to coffee leaf rust: A functional marker based approach
Source: PLoS One. 2020 Jul 8;15(7):e0222747. doi: 10.1371/journal.pone.0222747 (PMC7343155; doi:10.1371/journal.pone.0222747)

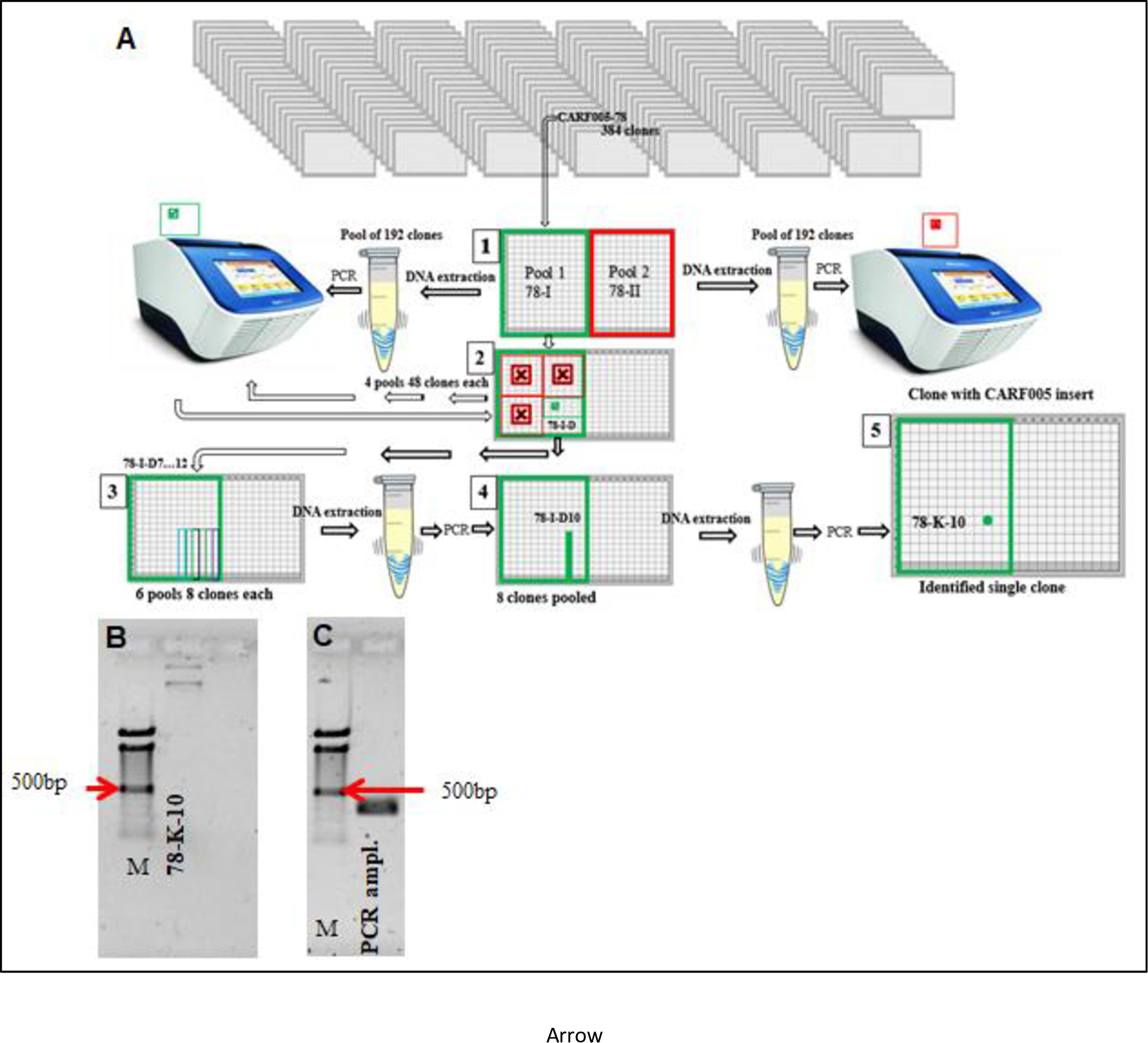

Supplement: S1 Fig — Clone pooling and subsequent group decomposition to isolate a single clone with CARF005 insert (A), DNA of isolated clone 78-K-10 (B) and CARF005 PCR amplicon (C) as revealed by 1% UltraPureTM agarose gel electrophoresis. M is 100 bp DNA size marker. The red arrow indicates the estimated size of marker DNA. (TIF) [file pone.0222747.s001.tif]

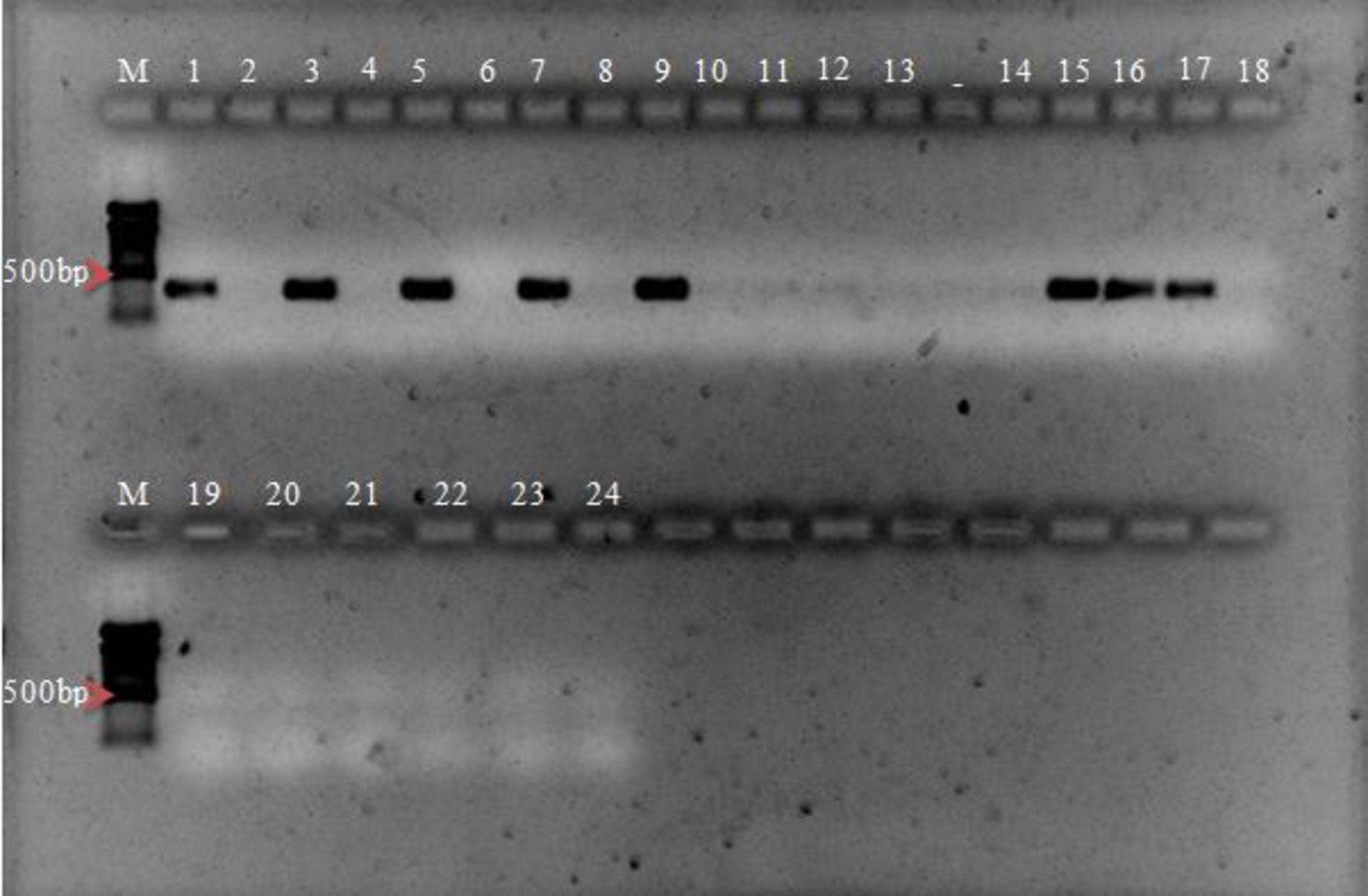

Supplement: S2 Fig — Clones with CARF005 were 1 (832/1-HT), 3 (1343/269-HT), 5 (H420/10), 7 (128/2-Dilla and Alghe), 9 (H419/20), 15 (4106), 16 (644/18 H. Kawisan, a new report) and 17 (832/2-HT). M: DNA weight marker ladder (the lightest band being 100 bp). The last three lanes (22–24) represent three coffee genotypes susceptible to all known races of H. vastatrix, used in this experiment as negative control for the CARF005 marker gene. The red arrow indicates the estimated size of marker DNA. No gel cropping was performed to any of the lanes displayed. (TIF) [file pone.0222747.s002.tif]

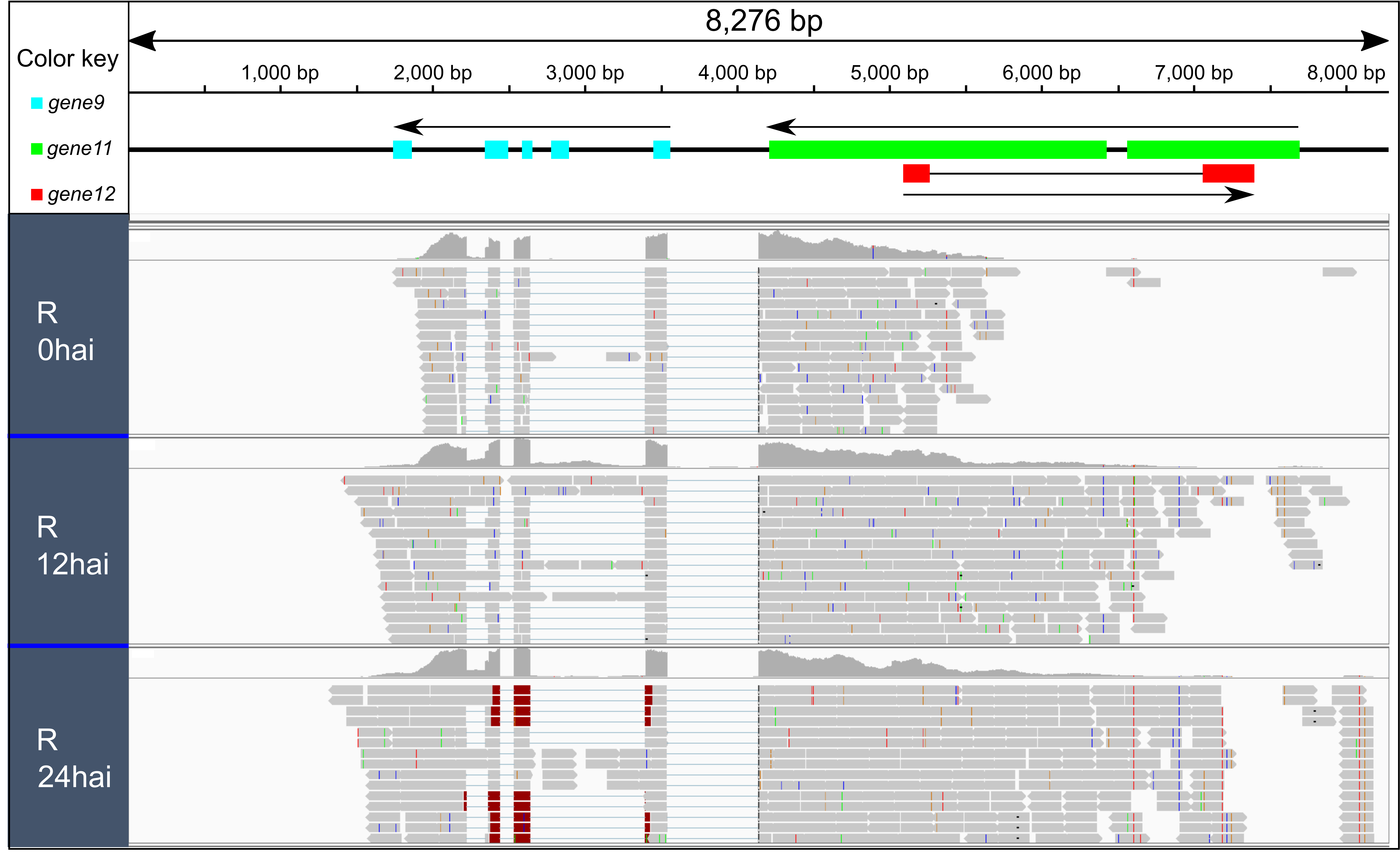

Supplement: S3 Fig — Note the three expression profiles (three rows) corresponding to control (uninoculated at 0 hour, top row), 12 (middle row) and 24 hai (bottom row) of transcriptome reads mapped against contig 9 of resistant coffee clone (CIFC HDT 832/2). Grey shades indicate matching transcriptome reads while nucleotide substitutions (mismatches) were shown by colored strips (yellow: G, green: A, red: R and blue: C). Large red shades indicate deletions. The three RGAs presented in different colors were selected due to their higher coverage. The contrasting difference in the differential expression was quite clear between the control sample (0hai) and the two samples taken at the other time points (12 & 24hai) and remarkable difference in the number of activated transcripts of the genes at 12 and 24hai. Contig mapping was performed by Tophat 228 (http://ccb.jhu.edu/software/tophat) setting alignment parameter as ‘-N 3—read-gap-length 3—read-edit-dist 6—no-coverage-search —b2-very-sensitive’ to locate the region of the contig encoding genes against the pathogen and visualized with Integrative Genomics Viewer (IGV) v. 2.3 [76] (http://www.broadinstitute.org/igv). (TIFF) [file pone.0222747.s003.tiff]
