# Supplementary material for: In silico guided structural and functional analysis of genes with potential involvement in resistance to coffee leaf rust: A functional marker based approach
Source: PLoS One. 2020 Jul 8;15(7):e0222747. doi: 10.1371/journal.pone.0222747 (PMC7343155; doi:10.1371/journal.pone.0222747)
Supplement: S1 Table — *Homologous sequences for which no ID/Accession number has been assigned are indicated in hyphen. BLASTp was performed by NCBI online server (https://blast.ncbi.nlm.nih.gov/Blast.cgi?PAGE=Proteins). (DOCX) [file pone.0222747.s004.docx]

| Gene (Query) | Top hit (species) | Subject ID/Accession numbers* | Description | E-value | Identity (%) |
| --- | --- | --- | --- | --- | --- |
| 1 | *Coffea arabica* | - | No description | 3.00E-176 | 79 |
| 1 | *Phyllostachys edulis* | [ADB85290.1](https://www.ncbi.nlm.nih.gov/protein/284434535?report=genbank&log$=protalign&blast_rank=1&RID=B8XUS9YU013) | Putative retrotransposon protein | 9.00E-94 | 54 |
| 2 | *Coffea canephora* | - | No description | 1.00E-26 | 76 |
| 3 | *Solanum lycopersicum* | [XP_010322277.1](https://www.ncbi.nlm.nih.gov/protein/723706811?report=genbank&log$=protalign&blast_rank=1&RID=3BXVH62B015) | PREDICTED: uncharacterized protein LOC104647985 | 1.00E-72 | 50 |
| 3 | *Coffea arabica* | - | No description | 1.00E-60 | 68 |
| 4 | *Coffea canephora* | [CDP13079.1](https://www.ncbi.nlm.nih.gov/protein/661883201?report=genbank&log$=protalign&blast_rank=1&RID=3BXVH62B015) | Unnamed protein product | 9.00E-05 | 91 |
| 5 | *Coffea canephora* | [CDP20136.1](https://www.ncbi.nlm.nih.gov/protein/661875372?report=genbank&log$=protalign&blast_rank=1&RID=3BXVH62B015) | Unnamed protein product | 0.0 | 91 |
| 5 | *Solanum tuberosum* | [XP_015160818.1](https://www.ncbi.nlm.nih.gov/protein/971586523?report=genbank&log$=protalign&blast_rank=75&RID=3BXVH62B015) | PREDICTED: putative disease resistance protein RGA4 | 0.0 | 38 |
| 6 | *Coffea canephora* | - | No description | 2.00E-73 | 68 |
| 7 | *Coffea canephora* | [CDP13085.1](https://www.ncbi.nlm.nih.gov/protein/661883207?report=genbank&log$=protalign&blast_rank=1&RID=3BXVH62B015) | Unnamed protein product | 0.0 | 98 |
| 7 | *Cynara cardunculus* | KVI07273.1 | Chloramphenicol acetyltransferase-like domain-containing protein | 2.00E-99 | 40 |
| 8 | *Coffea canephora* | - | No description | 2.00E-34 | 84 |
| 9 | *Coffea canephora* | [CDP13077.1](https://www.ncbi.nlm.nih.gov/protein/661883199?report=genbank&log$=protalign&blast_rank=1&RID=3BXVH62B015) | Unnamed protein product | 1.00E-35 | 67 |
| 9 | *Capsicum annuum* | [XP_016542041.1](https://www.ncbi.nlm.nih.gov/protein/1026018718?report=genbank&log$=protalign&blast_rank=3&RID=B8XUS9YU013) | PREDICTED: protein ALWAYS EARLY 3-like isoform X2 | 2.00E-10 | 58 |
| 10 | *Coffea canephora* | [GAQ44625.1](https://www.ncbi.nlm.nih.gov/protein/966762755?report=genbank&log$=protalign&blast_rank=1&RID=3BXVH62B015) | Unnamed RGA fragment | 5.00E-11 | 74 |
| 11 | *Coffea canephora* | CDP20093.1 | Unnamed protein product | 0.0 | 91 |
| 11 | *Solanum tuberosum* | [XP_015160818.1](https://www.ncbi.nlm.nih.gov/protein/971586523?report=genbank&log$=protalign&blast_rank=61&RID=B8XUS9YU013) | PREDICTED: putative disease resistance protein RGA4 | 0.0 | 37 |
| 12 | *Coffea canephora* | - | Putative disease resistance protein RGA3 complete | 1.00E-45 | 72 |
| 13 | *Coffea canephora* | - | No description | 4e-29 | 77 |
