# Supplementary material for: In silico guided structural and functional analysis of genes with potential involvement in resistance to coffee leaf rust: A functional marker based approach
Source: PLoS One. 2020 Jul 8;15(7):e0222747. doi: 10.1371/journal.pone.0222747 (PMC7343155; doi:10.1371/journal.pone.0222747)
Supplement: S2 Table — *As numbered from the first to the last residue, the hyphen indicates the amino acid (s) constituting the binding sites. Purple highlighted residues are conserved residues in both genes while yellow highlighted residues are specific protein binding sites in respective gene. Substitution mutation effect analysis was performed by The Predict Protein Server [31] (http://ppopen.rostlab.org). (DOCX) [file pone.0222747.s005.docx]

| Protein binding sites* | High effect | Moderate effect | No effect | Amino acid residues in the protein binding sites |
| --- | --- | --- | --- | --- |
| Gene 5 |  |  |  |  |
| 93-96 |  | X |  | YRNQ |
| 100 |  | X |  | K |
| 147 |  |  | X | K |
| 158 | X |  |  | R |
| 299 |  | X |  | T |
| 385 |  |  | X | K |
| 455-456 |  | X |  | RE |
| 463 |  |  | X | E |
| 527-530 |  | X |  | RRNK |
| 664 | X |  |  | K |
| 719-720 |  |  | X | QK |
| 750 |  |  | X | K |
| 790 |  |  | X | K |
| 821 | X |  |  | R |
| 1124 | X |  |  | R |
| Gene 11 |  |  |  |  |
| 93-95 |  |  | X | N |
| 143 |  | X |  | R |
| 416 |  |  | X | N |
| 429 |  |  | X | R |
| 729 | X |  |  | R |
| 1099-1100 |  |  | X | RR |
| 1111-1112 |  | X |  | QR |
