# Supplementary material for: In silico guided structural and functional analysis of genes with potential involvement in resistance to coffee leaf rust: A functional marker based approach
Source: PLoS One. 2020 Jul 8;15(7):e0222747. doi: 10.1371/journal.pone.0222747 (PMC7343155; doi:10.1371/journal.pone.0222747)
Supplement: S3 Table — *Ten contigs specific to C. arabica and three contigs specific to C. canephora, all assembled from BAC clones with SH3 locus were taken from the work of Ribas et al. [6]. (DOCX) [file pone.0222747.s006.docx]

| Query | Subject accession number | Identity (%) | Alignment length | Mismatch | Gap open | Query start | Query end | Subject start | Subject end | e-value | Bit score | Contig serial number |
| --- | --- | --- | --- | --- | --- | --- | --- | --- | --- | --- | --- | --- |
| contig3 | gb\|HQ696509.1\| | 88.247 | 485 | 56 | 1 | 11920 | 12403 | 124032 | 124516 | 4.33e-175 | 614 | *C. arabica*_7 |
| contig3 | gb\|GU123899.1\| | 88.795 | 473 | 52 | 1 | 11929 | 12400 | 208422 | 208894 | 5.28e-174 | 610 | *C. arabica*_6 |
| contig3 | gb\|HQ696513.1\| | 87.243 | 486 | 59 | 3 | 11920 | 12403 | 186601 | 187085 | 6.02e-167 | 587 | *C. canephora*_3 |
| contig3 | gb\|HQ696507.1\| | 87.243 | 486 | 59 | 3 | 11920 | 12403 | 13356 | 13840 | 6.02e-167 | 587 | *C. canephora*_1 |
| contig3 | gb\|GU123895.1\| | 72.468 | 385 | 69 | 15 | 12583 | 12953 | 101631 | 101992 | 2.41e-45 | 183 | *C. arabica*_2 |
| contig3 | gb\|GU123894.1\| | 70.698 | 430 | 63 | 18 | 12583 | 12965 | 47408 | 47821 | 2.41e-45 | 183 | *C. arabica*_1 |
| contig3 | gb\|HQ696512.1\| | 81.915 | 188 | 25 | 4 | 12583 | 12769 | 92911 | 92732 | 3.58e-43 | 176 | *C. canephora*_2 |
| contig3 | gb\|GU123898.1\| | 68.343 | 338 | 53 | 13 | 12583 | 12907 | 84162 | 83866 | 8.43e-26 | 118 | *C. arabica*_5 |
| contig3 | gb\|HQ696510.1\| | 73.913 | 207 | 36 | 7 | 12580 | 12786 | 120484 | 120296 | 1.03e-24 | 114 | *C. arabica*_8 |
| contig3 | gb\|GU123897.1\| | 64.972 | 354 | 64 | 11 | 12600 | 12931 | 93064 | 92749 | 1.74e-15 | 84.2 | *C. arabica*_4 |
| contig3 | gb\|HQ696511.1\| | 75.410 | 122 | 28 | 2 | 12283 | 12404 | 15692 | 15573 | 7.39e-14 | 78.8 | *C. arabica*_9 |
| contig3 | gb\|HQ696508.1\| | 87.755 | 49 | 6 | 0 | 12583 | 12631 | 45396 | 45444 | 5.68e-09 | 62.6 | *C. arabica*_10 |
| contig3 | gb\|GU123896.1\| | 80.303 | 66 | 13 | 0 | 12583 | 12648 | 100998 | 101063 | 1.98e-08 | 60.8 | *C. arabica*_3 |
| contig9 | gb\|GU123898.1\| | 77.647 | 170 | 38 | 0 | 7794 | 7963 | 258864 | 258695 | 1.57e-31 | 136 | *C. arabica*_5 |
| contig9 | gb\|HQ696508.1\| | 76.536 | 179 | 38 | 4 | 7771 | 7946 | 68305 | 68482 | 1.21e-26 | 120 | *C. arabica*_10 |
