# Supplementary material for: In silico guided structural and functional analysis of genes with potential involvement in resistance to coffee leaf rust: A functional marker based approach
Source: PLoS One. 2020 Jul 8;15(7):e0222747. doi: 10.1371/journal.pone.0222747 (PMC7343155; doi:10.1371/journal.pone.0222747)
Supplement: Attachment — Submitted filename: Response to reviewers.docx [file pone.0222747.s007.docx]

**Response to reviewers’ comments**: **green** font color

**Reviewer #1:** The authors have described an important study relating to the disease resistance genes against coffee rust. The identification of two groups of RGAs, the NBS-LRR & non-NBS-LRR contained on the SH loci demonstrate polymorphism which is a new insight into molecular characterization of these genes.

R: Thank you.

**Reviewer #2:** The coffee is a cash crop and widely grown in different parts of the world. Coffee leaf rust (CLR) caused by an obligate fungal parasite Hemileia vastatrix is one of the major diseases costing millions of dollar loss to coffee growers. Identification of resistance gene and its utilization in the breeding program will be a substantial contribution to the coffee research. Here Authors have made an effort to identify resistance gene(s) for H. vastatrix by screening the BAC clone library of the resistance coffee crop, Hibrido de Timor (HDT), using the CARF005 marker. It is an innovative approach to identify resistance genes in coffee and hence worth pursuing. However, in the process of achieving the goal, the authors have fallen short at several points.

Major concerns:
• Authors have mentioned supplementary figure S5 in line no 273, Table S6 in line no 315, and table no S7 in line no 338, but these figures and tables were not provided to the reviewer to review.

R: Changes were made to figure and table numbers. Accordingly, figure ‘S5 Fig.’ was changed to ‘S4 Fig.’, ‘S6 Table’ to ‘S2 Table’ and ‘S7 Table’ to ‘S3 Table’. Moreover, the citation of S1 Table was introduced at the right place in the text.

• Also, the Authors mentioned about generating RNA-Seq libraries and its sequencing in line no 112 in material method sections. But detailed method of the RNA-Seq libraries preparation and sequencing are not provided anywhere. Authors need to confirm that if they have generated new RNA-Seq libraries for this study or cross-referenced to Florez et al., 2017. if authors have performed RNA-Seq for this study, then they should submit the sequences to NCBI SRA and provide the accession number here.

R: The generation of RNA-seq was conducted by Florez et al., 2017 (also conducted in our lab). The reference is now properly cited in the text. The transcriptome library was used for our study in an effort to map the differentially expressed transcripts in response to the *H. vastatrix* (**race XXXIII)** to the identified contig (contig 9) with the candidate RGAs. As the work was published (with seq data available in data repository), the details of library preparation and sequence assembly could be referred from the work of Florez et al., 2017. We made changes in the text to make clearer that these datasets came from Florez et al., 2017. Also, here are the links to the RNA-seq (SRA and other resources) of the report in NCBI database.

Links to Bioprojects:

- PRJNA353233: Coffea arabica x Coffea canephora Raw RNAseq reads (Hibrido de timor) <https://www.ncbi.nlm.nih.gov/bioproject/PRJNA353233>
- PRJNA353185: Coffea arabica cv caturra raw RNAseq reads (Caturra) <https://www.ncbi.nlm.nih.gov/bioproject/PRJNA353185>

Links to SRAs:

- SRP103086: Coffea arabica x Coffea canephora Raw RNAseq reads (Hibrido de timor) <https://www.ncbi.nlm.nih.gov/sra/?term=SRP103086>
- SRP103087: Coffea arabica cv caturra raw RNAseq reads (Caturra) <https://www.ncbi.nlm.nih.gov/sra/?term=SRP103087>

• The authors mentioned about the differential expression of gene 11 upon H.vastatrix infection based on RNA-Seq data but did not provide any supporting data to substantiate the statement. The authors need to give the fold changes and p-value from the RNA-Seq data for all the putative genes identified in this study, and further confirm it by performing qRT-PCR.

R: The differential expression analysis in this work was an indirect approach by using the differentially upregulated transcripts exclusive to renowned rust resistant Hibrido de Timor clone CIFC 832/2, based on the RNA-seq (Florez et al. 2017) profile in response to the target pathogen at 12 and 24 hai. The differential expression of candidate RGAs (including gene 11) spanning contig 9 was clearly visible by comparing the map of the transcriptome data against contigs 9, which resulted in the generation of differentially expressed transcripts as shown in S4 Fig (gray shades, at two time points, 12 & 24hai). The confirmation for differential expression was evident, as no such map with contrasting expression profile at the two time points was possible against contig 3 (map was not possible to generate). The reviewer is right in requesting for the p-value of the fold changes and the requirement of differential expression analysis by qRT-PCR, as it is the most common and routine method of differential expression validation. The differential expression of the sequenced transcripts used to map to contig 9 was confirmed by Florez et al. 2017, first by resistance gene transcript reads filtration and then transcript read assembly using *C. canephora* genome as reference and *de novo* assembly. Subsequently, qRT-PCR was used to validate some of the mapped transcripts (13 RGAs) exclusively activated during the incompatible interaction. Since the assembled transcriptome was proved to harbor transcripts (exons) activated (differentially expressed) in response to the inoculation of the pathogen, our main target was to confirm that our contig (contig 9, containing the target RGAs, including gene 11) harbors genes differentially expressed (variants of resistance gene transcripts/exons) in response to *H. vastatrix* (**race XXXIII)** inoculation. We believe in the importance of qRT-PCR and other biological resistance phenotype investigations for further complementation, but our mapping strategy could also be considered as the best *in silico*-based alternative.
• The title is not appropriately reflecting the work done in the manuscript. Most of the structural and functional prediction is the manuscript is done in silico. Thereby, simple mentioning structural and functional analysis will mislead the readers.The author should add in silico to the title to make it clear to the reader.

R: The title has been modified as suggested.
• Most of the figure legends lack essential information to understand the figure. For example, Figure 1 legend provides the least information. While the title of the figure says about the comparison of the conserved domains, the image is just a snapshot of the NCBI BLAST. And, the red circling does not provide any information about the polymorphism. In Figure 2, the authors mentioned that the protein binding region of gene 5 is boxed in red; instead, they used red underline to mark the region. Same for the gene 11 and green boxes. Authors failed to mention how they come to know that these are the conserved protein binding regions, and based on what? Figure legends should carry enough information about the figure that readers can understand it without digging into the main text.

R: Legends for the two figures (now Figures 2 and 3) were modified. The blue encircled regions shown on the two genes’ alignment in Figure 3 were conserved protein regions based on the protein prediction analysis (PredictProtein online tool) (Yachdav et al., 2014, URL: <https://open.predictprotein.org/>). S2 Table was cited in Figure 3 legend with further explanation for more clarification and understanding of the data on which the authors were based to spot the conserved protein binding regions.

• Some portions of the material and method section are poorly written. For example, the details about how the phylogenetic tree is generated for figure 4 is not mentioned in the material method section but nicely presented in figure legends. Similarly, authors failed to provide detail information about how the 3D structures of genes 5 and 11 were generated other than merely mentioning the software used for this. Information such as the PDB template used to predict the 3D structure is crucial along with the parameters used for energy minimization. The author should provide enough information about the methods used such that readers can able to replicate the work.

R: Modifications were made to some sections under ‘materials and methods’. Functional and phylogenetic analysis sub-section under ‘Materials and methods’ was revised and modified for better understanding of the conducted analysis (for easy understanding of Figure 4, now Figure 5, later in the results and discussion sections as a reader goes down reading the manuscript). Also, modifications were made to ‘Gene prediction and annotation’ sub-section of the ‘Materials and methods’ section.
• The authors need to provide synteny maps for the comparison of putative genes with the Canephora and arabica genome.

R: Synteny map was generated for the putative RGAs (genes 9, 10, 11 and 12) by using *C. canephora* and *C. arabica* genomes and included as Fig 1.

Minor concerns:
• In Suppl fig. S4, authors need to mark the gene 9-12.

R: Suppl. Fig S4 marked to show the differential expression of RGAs at different time points following pathogen inoculation. Modification was also made to the legend of Suppl. Fig 4 based on the edition to the figure (rectangular mark of the region with active transcription of R genes in red color).
• Inline 270-271, the authors mentioned about two contigs mapped against the transcriptome but only provide images for the contig 9 in suppl fig S4 and wrongly mentioned it fig S5.

R: Yes, the transcriptome was mapped against the two contigs (3 & 9) separately. However, no map was possible to generate when the transcriptome data was mapped against contig 3 unlike the one performed against contig 9 (S4 Fig). That was the reason as to why only the map generated when contig 9 was used was provided. The wrongly mentioned S5 Fig was corrected as S4 Fig.
• Inline no 190, authors have mentioned that they have tested 21 differential coffee clones using marker CARF005. However, in the FigS2, the gel contains 24 lanes. The authors did not say anything about lane no 22-24 both in figure legends and main text but mentioned it in table 1. The consistency should be maintained. Also, the authors need to label the markers in the gel and specify the size of the PCR band. Same for the gel images in Suppl Fig1b and c.

R: Description was added to S2 Fig legend. The main text under the results section was also revised (and modified) to include the information about the three last lanes on the gel. Also, modifications to the S1 Fig (b & c) and S2 Fig and their legends were made.
