# Supplementary material for: In silico guided structural and functional analysis of genes with potential involvement in resistance to coffee leaf rust: A functional marker based approach
Source: PLoS One. 2020 Jul 8;15(7):e0222747. doi: 10.1371/journal.pone.0222747 (PMC7343155; doi:10.1371/journal.pone.0222747)
Supplement: Attachment — Submitted filename: Response to reviewers.docx [file pone.0222747.s008.docx]

**Point-by-point responses to the reviewers’ comments (responses in green font color):**

Reviewer #1: 1. The authors have addressed the earlier comments. The revised version also includes the supplemental figures.

Reviewer #2: The authors have made an effort to address the questions raised in the first review. However, there are still some grievances that need to be addressed.

Major concerns:
1) The results sections are poorly written, lacking the rationale for the experiment and also the clarity of the experiment. Such as why authors decided to do the gene annotation when they already mentioned genes 5 and 11 are RGAs.

- R: Gene annotation analysis was performed for the two target RGAs (genes 5 and 11) for further characterization in terms of the associated gene ontology (GO) terms, basically ; molecular function, biological process and cellular component in order to reveal their resistance specificity and cellular localization. Gene annotation section has been modified as per the suggestion.

2) And in some cases, there is nothing mentioned about the figure in the result section. For example, nothing is said about figure 3 in the result section.

- R: We agree on the need of mentioning (some description) of the figures cited in the text. Further mentioning was made for Figure 3 in addition to the two times citation of the figure in the results section.

3) Poor choice of the software for the study. For example, for identifying the conserved domain, the authors used the blastp program instead of the conserved domain database (CDD) of NCBI or similar databases such as SMART that are widely used for this purpose.

- R: The online tool applied for conserved protein domain search was wrongly cited as ‘BLASTp of the NCBI ((<https://blast.ncbi.nlm.nih.gov/Blast.cgi?PAGE=Proteins>)’ despite the fact that the actually used tool was protein Conserved Domain Database (CDD) of the NCBI (<https://www.ncbi.nlm.nih.gov/Structure/cdd/wrpsb.cgi>). Figure 2 legend was modified accordingly. We are sorry for the wrong reference made.

4) Poor presentation of the figure. Such as
a. For example, take suppl fig3. It is hard to understand what the authors are trying to share. In figure legend, the authors mentioned the black arrow shows the orientation. In the figure, it is hard to locate the black arrows for gene 11,12 and 10. What is the orange box represents? Again, the poor choice of software/program.

- R: The purpose of including Suppl. Fig 3 was to demonstrate the location, arrangement and trace homologs of the four RGAs (genes 9, 10, 11 and 12) in the genome of *C. canephora*. As there is no other option (other software/online tool) than the *C. canephora* genome hub for such analysis, we tried to reveal the position, arrangement and homolog RGAs by BLASTn and then by Gbrowse in the genome hub but unfortunately, it was impossible to enhance the quality of the generated mapping figure. It could be recalled that we introduced synteny map in our last revision, which is a redundancy of Suppl. Fig 3 in its content, hence including Suppl.Fig3 is not important as the information conveyed in this figure is already contained in Fig 1 and S1Table. Therefore, we excluded Suppl. Fig 3 from the manuscript, re-numbered the supplemental figures and edited the Suppl. Fig citations in the entire manuscript.

b. In figure 2, the authors failed to mention which one is gene 5 and which one is gene 11.

- R: New graphic illustrations were generated with some new and up-to-date conserved domain features for both RGA proteins [Note: some differences (new features) in the domains of the latest version are believed to be attributed to the new data being fed into the NCBI CDD]. Also, the figures have been labeled with proper genes as per the suggestion.

c. Figure 3: Multiple sequence alignment amino acids were color-coded, which is unnecessary and it makes the underling of amino acid hard to track.

- R: The figure and its legend have been improved, with no colored amino acids now.

d. The figure quality is not good and pixelated. For example, figure 4 top numbering is hard to follow.

- R: The quality of Figure 4 has been improved. The top numbering has now been excluded as the residues of the protein binding sites (spikes) for both gens has been listed (along with point mutation effect prediction for the constituent residues) in Supplemental Table 2. The number on the top right is just to mention the size of the proteins.

5. Suppl figure4: Authors tried to show the differential expression of genes of contig 9 compared control.
a) The image is of poor quality with hard to read the numbers on top.

- R: The figure was edited for better presentation.

b) b. Authors, instead of approximately drawing a rectangle around the gene cluster, should have shown the exact position of the genes.

- R: Gene positions were included in the figure

c) c. Authors also need to provide information about how many transcripts read hit to the genes at different experimental conditions (control vs. different time points) with fold change and p-value in this manuscript instead of directing the reader to their previous publication.

- R: As we were using only the contigs as references, a software to identify and quantify differentially expressed genes, such as DESEq2, cannot be used, as the reference is very short (contigs). Number of aligned reads is crucial for differential analysis in RNAseq experiments, therefore, there is no statistical power allowing the software to calculate differential expression using a minimal number of reads to map against the reference contig. With this limitation in mind, we performed a qualitative analysis, based on the number of reads mapping against these genes (contig), as shown in S3 Fig, which show that at 0h, only two reads map in the position, while in latter time points (12hai and 24hai) it is possible to see reads pilling up in the same position, indicating that these genes are expressed after inoculation. We changed the text to make it clear that it was only a qualitative analysis.

d) Similarly, authors should provide the transcript reads that are matched to the gene 5 of contig three and let readers have that information instead of taking the author's word for granted.

- R: Unlike the mapping of the transcriptome against contig 9, mapping against contig 3 did give no reads matching gene 5. Therefore, no graphic illustration of matching reads as was the case against contig 9 was possible to generate.

6. The authors hardly cited the figures in their discussion section. Also, the authors failed to discuss what they things could be the reason for gene 11 differentially expressed but not gene 5 when both are RGAs.

- R: More figure and table citation has been included. Based on our results and the current understanding of the molecular mechanism of plant defense, the possible reasons for the differential expression of gene 11 (but not gene 5) in response to the attack by *H. vastatrix* has been discussed (Lines 419-433).

Minor concerns:
1. Inline no 113: nanodrop generally provides the quantity and quality of the nucleotides and not the integrity. It needs to be corrected.

- R: The sentence has been corrected.

2. Authors, in some cases, provided the link to the software they have used in this study and some cases not. Consistency needs to be followed.

- R: The links of the software/online tools used were now included.

3. Inline no 273: authors said CARF005 amplified the region 2065 to 3115 of gene 11, which is of 1050 bps long while the PCR product is around 400 bps. It needs to be corrected.

- R: Corrected.

4. The figure legend of figure 5 is more about how the figure generated rather than what the figure signifies.

- R: The figure legend has been modified.
